# GCN5 modulates salicylic acid homeostasis by regulating H3K14ac levels at the 5′ and 3′ ends of its target genes

**DOI:** 10.1093/nar/gkaa369

**Published:** 2020-05-12

**Authors:** Soonkap Kim, Sophie J M Piquerez, Juan S Ramirez-Prado, Emmanouil Mastorakis, Alaguraj Veluchamy, David Latrasse, Deborah Manza-Mianza, Rim Brik-Chaouche, Ying Huang, Natalia Y Rodriguez-Granados, Lorenzo Concia, Thomas Blein, Sylvie Citerne, Abdelhafid Bendahmane, Catherine Bergounioux, Martin Crespi, Magdy M Mahfouz, Cécile Raynaud, Heribert Hirt, Vardis Ntoukakis, Moussa Benhamed

**Affiliations:** 1 Institute of Plant Sciences Paris-Saclay (IPS2), UMR 9213/UMR1403, CNRS, INRA, Université Paris-Sud, Université d’Evry, Université Paris-Diderot, Sorbonne Paris-Cité, Bâtiment 630, 91405 Orsay, France; 2 Division of Biological and Environmental Sciences and Engineering, King Abdullah University of Science and Technology, Thuwal 23955-6900, Kingdom of Saudi Arabia; 3 School of Life Sciences and Warwick Integrative Synthetic Biology Centre, University of Warwick, Coventry CV4 7AL, UK; 4 Institut Jean-Pierre Bourgin, INRA, AgroParisTech, CNRS, Université Paris-Saclay, Versailles 78000, France; 5 Institut Universitaire de France (IUF)

## Abstract

The modification of histones by acetyl groups has a key role in the regulation of chromatin structure and transcription. The *Arabidopsis thaliana* histone acetyltransferase GCN5 regulates histone modifications as part of the Spt-Ada-Gcn5 Acetyltransferase (SAGA) transcriptional coactivator complex. GCN5 was previously shown to acetylate lysine 14 of histone 3 (H3K14ac) in the promoter regions of its target genes even though GCN5 binding did not systematically correlate with gene activation. Here, we explored the mechanism through which GCN5 controls transcription. First, we fine-mapped its GCN5 binding sites genome-wide and then used several global methodologies (ATAC-seq, ChIP-seq and RNA-seq) to assess the effect of GCN5 loss-of-function on the expression and epigenetic regulation of its target genes. These analyses provided evidence that GCN5 has a dual role in the regulation of H3K14ac levels in their 5′ and 3′ ends of its target genes. While the *gcn5* mutation led to a genome-wide decrease of H3K14ac in the 5′ end of the GCN5 down-regulated targets, it also led to an increase of H3K14ac in the 3′ ends of GCN5 up-regulated targets. Furthermore, genome-wide changes in H3K14ac levels in the *gcn5* mutant correlated with changes in H3K9ac at both 5′ and 3′ ends, providing evidence for a molecular link between the depositions of these two histone modifications. To understand the biological relevance of these regulations, we showed that GCN5 participates in the responses to biotic stress by repressing salicylic acid (SA) accumulation and SA-mediated immunity, highlighting the role of this protein in the regulation of the crosstalk between diverse developmental and stress-responsive physiological programs. Hence, our results demonstrate that GCN5, through the modulation of H3K14ac levels on its targets, controls the balance between biotic and abiotic stress responses and is a master regulator of plant-environmental interactions.

## INTRODUCTION

Histone-modifying enzymes add or remove covalent histone modifications that alter the accessibility of eukaryotic DNA to transcription factors, mediating the dynamic transition between expressed and repressed genomic regions (1). Different histone and DNA modifications are generally associated with a specific transcriptional state. For instance, acetylation marks and methylations of lysine 4 of histone 3 (H3K4ac, H3K4me3 and H3K4me1) are linked to transcriptionally active genes (2–4), whereas the dimethylation of lysine 9 (H3K9me2) and trimethylation of lysine 27 (H3K27me3) are associated with transcriptional repression (5–7).

The four core eukaryotic histone proteins can be acetylated and deacetylated on diverse residues of their N-terminal tails, giving rise to a plethora of putative acetylation sites on a single nucleosome (8). Histone acetylation appears to physically alter chromatin conformation by reducing the affinity between histones and DNA, allowing the recruitment of the transcriptional machinery in *cis* (9,10). The levels of these histone modifications are modulated throughout development and in response to environmental cues through the activity of histone acetyltransferases (HATs) and deacetylases (HDACs), which deposit and remove acetyl groups from histones, respectively (2,3,8,11,12). The *Arabidopsis thaliana* genome encodes 12 HATs that are classified into two classes according to their cellular location: Type A HATs localize in the nucleus and acetylate nucleosomal histones, while Type B HATs localize in the cytoplasm and catalyze the acetylation of free histones (13). Type A HATs are divided into four families: MYST, p300/CBP, TAF1 and GCN5-related *N*-acetyltransferases (GNATs) (13,14). GNATs contain an N-terminal HAT-domain and a C-terminal bromodomain, considered to be a targeting motif (15,16).

AtGCN5/HISTONE ACETYLTRANSFERASE GCN5 1 (HAG1) is a well-studied GNAT that participates in the Spt-Ada-Gcn5 Acetyltransferase (SAGA) complex, a transcriptional coactivator involved in various physiological programs through the regulation of histone modifications. In yeast, this complex has been extensively characterized and described as a regulator of the expression of ∼10% of the genome, with an enrichment in stress-related genes (17). SAGA regulates gene expression in yeast through diverse mechanisms, including histone acetylation via GCN5 activity (18), histone deubiquitination (19), regulation of the basal transcription machinery (20) and mRNA export from the nucleus (21). Despite the abundant genetic information and tools, several aspects of the composition and function of the SAGA complex in plants remain obscure (22).

*Arabidopsis* GCN5 participates in the histone acetylation module of the SAGA complex, together with ADA2, ADA3 and SGF29 (22). Since it contains a HAT domain and a bromodomain, GCN5 is considered to be both a reader and a writer of histone acetylation. GCN5 acetylates lysine 14 of histone 3 (H3K14ac) in promoter regions of its targets, and influences H3K9ac and H3K27ac levels (14,23,24); however, the mechanism by which it controls transcription remains unknown. GCN5 is involved in several developmental processes and responses to environmental stimuli. Indeed, the *gcn5* mutation leads to a pleiotropic developmental phenotype that includes dwarfism, as well as aberrant organ development and flower organ identity (25–30). Furthermore, GCN5 participates in the control of iron homeostasis, the accumulation of cuticular wax, and the regulation of responses to different abiotic stimuli, such as light, cold and heat (23,31–35).

Through a chromatin immunoprecipitation (ChIP)-on-chip approach, we previously showed that, in general, GCN5 is a positive regulator of gene expression (36), as expected for a HAT. However, we observed that GCN5 binding did not systematically correlate with gene activation (36). To gain further insight into the molecular function of GCN5, in this study, we applied several genome-wide technologies to finely map GCN5 binding sites and impact on the epigenetic regulation and expression of its target genes. This analysis provided evidence for a dual role of GCN5 in the regulation of H3K14ac levels in 5′ and 3′ ends of its target genes in addition to promoters. Furthermore, we demonstrated a correlation between H3K14ac and H3K9ac levels at both 5′ and 3′ ends pointing at a molecular link between the depositions of these two histone modifications. Finally, through target analysis and physiological measurements, we demonstrated that GCN5 acts as an integrator of responses to environmental stimuli in the crosstalk between developmental and stress-responsive physiological programs.

## MATERIALS AND METHODS

### Plant material and growth conditions

All plant materials used in this study were in *Arabidopsis thaliana* ecotype Columbia (Col-0) background (referred hereafter as WT). The following T-DNA insertion mutants and transgenic plants were used: *gcn5-1* (SALK_106557C), *gcn5-2* (SALK_150784C), *sid2-1* (37) and *NahG* (38). Two different lines expressing *GCN5* in *gcn5* mutant background were generated. First, we amplified the coding region of *GCN5* (At3g54610) from total WT cDNAs using the following primers: forward, 5′-ACGC**GTCGAC**ATCCACTCTCACTCTTCCCACCT-3′ and reverse, 5′-TATCAAAT**GCGGCCGC**TTGAGATTTAGCACCAG-3′. The PCR fragment was digested with SalI and NotI restriction enzymes (whose sites are highlighted in bold) and inserted into pENTR1A, before being recombined into pB7FWG2,0. This cloning gave the *35S::GCN5-GFP* construct. Second, we amplified *GCN5* from WT gDNA, including 719 bp of *GCN5* promoter, using the following primers: forward, 5′- **AAAAAGCAGGCTCCACC**TGTCAAGTGGTGCTTTAAC-3′ and reverse 5′- **AGAAAGCTGGGTC**TTGAGATTTAGCACCAG-3′ (containing mini-attB sites highlighted in bold). The PCR fragment was further amplified using full attB1/attB2 primers: forward, 5′- GGGGACAAGTTTGTACAAAAAAGCAGGCT-3′ and reverse 5′- GGGGACCACTTTGTACAAGAAAGCTGGGT-3′. The PCR fragment was then subcloned into pDONR/Zeo before being recombined into pEarleyGate302. This cloning gave the *GCN5p::GCN5-FLAG* construct. The final vectors were transformed into *Agrobacterium tumefaciens* strain GV3101 by electroporation. *A. thaliana gcn5* mutant plants were transformed by floral dip method (39), *gcn5-1* was transformed with *35S::GCN5-GFP* construct, while *gcn5-2* was transformed with *GCN5p::GCN5-FLAG* construct. Transformants were selected on phosphinothricin (20 μg/mL). Plants were grown *in vitro* in controlled environment chambers on sterile half-strength MS medium containing 0.8% agar under long day conditions (16h of light at 20°C, 8 h of darkness at 18°C). Seeds were surface-sterilized by treatment with diluted bleach for 10 min, washed, and imbibed in sterile-water for 2–4 days at 4°C to obtain homogeneous germination. Adult plants were grown in short day conditions (8 h of light at 20°C, 16 h of darkness at 18°C).

### *Pseudomonas syringae* disease assay

*Pseudomonas syringae* pv. *tomato* DC3000 (*Pst* DC3000) was used for infection experiments. *Pst* DC3000 cultures were grown at 28 °C on selective media (King's B supplemented with 50 μg/ml rifampicin). On the day of the infection, overnight cultures were centrifuged, washed and resuspended in 10 mM MgCl_2_. The bacterial suspension was adjusted to OD600 = 0.2, to which 0.04% Silwet-L-77 were added. 5-week-old plants were sprayed with the *Pst DC3000* suspension until completely covered and incubated for 3 days in a saturating humidity environment. For the determination of bacterial population, leaf disks were collected for each genotype, washed with 70% ethanol and sterile water, and ground in 10 mM MgCl_2_. The resulting bacterial suspension was serially diluted 1:10 and plated on selective media. Bacterial populations were determined per leaf area 0- and 3-days post inoculation.

### Salicylic acid quantification

5-week-old rosettes were collected, frozen in liquid nitrogen, and lyophilized until completely dehydrated. Dehydrated tissue was ground to a fine powder, and for each sample, 3 mg of dry powder were extracted with 0.8 mL of acetone/water/acetic acid (80/19/1 v:v:v). SA stable labelled isotopes used as internal standards were prepared as described previously (40). One ng of each standard was added to the sample. The extract was vigorously shaken for 1 min, sonicated for 1 min at 25 Hz, shaken for 10 min at 10°C in a Thermomixer (Eppendorf^®^, and then centrifuged (8000 g, 10°C, 10 min). The supernatants were collected, and the pellets were re-extracted twice with 0.4 ml of the same extraction solution, then vigorously shaken (1 min) and sonicated (1 min; 25 Hz). After the centrifugations, the three supernatants were pooled and dried (final volume 1.6 ml). Each dry extract was dissolved in 100 μl of acetonitrile/water (50/50 v/v), filtered, and analyzed using a Waters Acquity ultra performance liquid chromatograph coupled to a Waters Xevo Triple quadrupole mass spectrometer TQS (UPLC–ESI-MS/MS). The compounds were separated on a reverse-phase column (Uptisphere C18 UP3HDO, 100 × 2.1 mm × 3 μm particle size; Interchim, France) using a flow rate of 0.4 ml min^−1^ and a binary gradient: (A) acetic acid 0.1% in water (v/v) and (B) acetonitrile with 0.1% acetic acid, the column temperature was 40°C. We used the following binary gradient (time, % A): (0 min, 98%), (3 min, 70%), (7.5 min, 50%), (8.5 min, 5%), (9.6 min, 0%), (13.2 min, 98%), (15.7 min, 98%). Mass spectrometry was conducted in electrospray and Multiple Reaction Monitoring scanning mode (MRM mode) in negative ion mode. Relevant instrumental parameters were set as follows: capillary 1.5 kV (negative mode), source block and desolvation gas temperatures 130°C and 500°C, respectively. Nitrogen was used to assist the cone and desolvation (150 l h^−1^ and 800 l h^−1^, respectively), and argon was used as the collision gas at a flow of 0.18 ml min^−1^. For each genotype three technical replicates were performed.

### RNA-seq assay

Total RNAs were extracted from 180 mg of the aerial part of seedlings with the ZR Plant RNA MiniPrep kit (Zymo Research), according to the manufacturer's instructions. HiSeq 50 bp singleton reads from RNA-Seq were first adaptor trimmed and then analyzed using the TopHat and Cufflinks software. TopHat (v2.0.9) was used for alignment of short reads to the *Arabidopsis thaliana* genome TAIR10, Cufflinks (v2.2.0) for transcript assembly and differential expression, and commeRbund (v2.0.0) for visualization of differential analysis. Default parameters were used. A total of three biological replicates were generated for the WT and *gcn5* RNA-seq data.

### Chromatin Immunoprecipitation with high-throughput sequencing (ChIP-seq) assay

ChIP-seq assays were performed on the aerial part of *in vitro* grown 14-day-old seedlings using anti-GFP (Clontech 632592), anti-H3K9ac (Millipore 07-352), anti-H3K14ac (Millipore 07-353), anti-H3K27me3 (Millipore 07-449), following a procedure modified from Gendrel *et al.* (41). Five grams of seedlings were cross-linked in 1% (v/v) formaldehyde at room temperature for 15 min. Crosslinking was then quenched with 0.125 M glycine for 5 min. The crosslinked seedlings were ground, and nuclei were isolated and lysed in Nuclei Lysis Buffer (1% SDS, 50 mM Tris–HCl pH 8, 10 mM EDTA pH 8). Cross-linked chromatin was sonicated using a water bath Bioruptor UCD-200 (Diagenode, Liège, Belgium) (15 s on/15 s off pulses; 15 times). The complexes were immunoprecipitated with antibodies overnight at 4°C with gentle shaking and incubated for 1 h at 4°C with 40 μl of Protein AG UltraLink Resin (Thermo Scientific). The beads were washed 2 × 5 min in ChIP Wash Buffer 1 (0.1% SDS, 1% Triton X-100, 20 mM Tris-HCl pH 8, 2 mM EDTA pH 8, 150 mM NaCl), 2 × 5 min in ChIP Wash Buffer 2 (0.1% SDS, 1% Triton X-100, 20 mM Tris–HCl pH 8, 2 mM EDTA pH 8, 500 mM NaCl), 2 × 5 min in ChIP Wash Buffer 3 (0.25 M LiCl, 1% NP-40, 1% sodium deoxycholate, 10 mM Tris–HCl pH 8, 1 mM EDTA pH 8) and twice in TE (10 mM Tris–HCl pH 8, 1 mM EDTA pH 8). ChIPed DNA was eluted by two 15-min incubations at 65°C with 250 μl Elution Buffer (1% SDS, 0.1 M NaHCO_3_). Chromatin was reverse-crosslinked by adding 20 μl of NaCl 5 M and incubated over-night at 65°C. Reverse-crosslinked DNA was submitted to RNase and proteinase K digestion and extracted with phenol-chloroform. DNA was ethanol precipitated in the presence of 20 μg of glycogen and resuspended in 50 μl of nuclease-free water (Ambion) in a DNA low-bind tube. 10 ng of IP or input DNA was used for ChIP-Seq library construction using NEBNext^®^ Ultra DNA Library Prep Kit for Illumina^®^ (New England Biolabs) according to manufacturer's recommendations. For all libraries, twelve cycles of PCR were used. The quality of the libraries was assessed with Agilent 2100 Bioanalyzer (Agilent). In total, several independent H3K14ac ChIP-seq experiments were performed, two for WT, three for the *gcn5-2* mutant and one for the *gcn5-1* mutant. H3K9ac and H3K27me3 ChIP-seq were performed twice independently. GCN5 ChIP-seq was performed twice independently on the *gcn5-1 35S::GCN5-GFP* complementation line.

### Computational analysis of ChIP-seq

Single-end sequencing of ChIP samples was performed using Illumina NextSeq 500 with a read length of 76 bp. Reads were quality controlled using FASTQC (http://www.bioinformatics.babraham.ac.uk/projects/fastqc/). Trimmomatic was used for quality trimming. Parameters for read quality filtering were set as follows: Minimum length of 36 bp; Mean Phred quality score greater than 30; Leading and trailing bases removal with base quality <5. The reads were mapped onto the TAIR10 assembly using Bowtie (42) with mismatch permission of 1 bp. To identify significantly enriched regions, we used MACS2 (43). Parameters for peaks detection were set as follows: Number of duplicate reads at a location:1; mfold of 5:50; *q*-value cutoff: 0.05; extsize 200; broad peak. Visualization and analysis of genome-wide enrichment profiles were done with IGB. Peak annotations such as proximity to genes and overlap on genomic features such as transposons and genes were performed using BEDTOOLS INTERSECT. SeqMINER was used for quantitative clustering based on tag density using a Density Array method with a wiggle window of 50 bp. NGSplot was used to profile the enrichment of this mark at transcriptional start sites (TSSs) and along the gene (44). To identify regions that were differentially enriched in the H3K14ac histone modification between WT and *gcn5* mutants, we used DIFFREPS (45) with parameters of *P*-value 0,05; *z*-score cutoff 2; windows 1000; G-test statistical test for H3K14ac. *De novo* motif analysis of GCN5 binding regions were screened using HOMER (46).

### ChIP-qPCR assay

ChIP-qPCR experiments were used to highlight specific GCN5 target genes identified from the ChIP-seq results and were performed on the two *gcn5* complemented lines, *gcn5-1 35S::GCN5-GFP* and *gcn5-2 GCN5p::GCN5-FLAG*. ChIP-qPCR assays were performed on 14-day-old seedlings and the chromatin-protein complexes isolation was performed as described by Ramirez-Prado *et al.* (47) with slight modifications: an initial double crosslink with 25 mM EGS (ThermoFisher Scientific, 21565) and 1% formaldehyde was performed on ground frozen material; the crosslinked chromatin was therefore sonicated longer until it reached an average 400bp length; immunoprecipitations used GFP-Trap Magnetic Agarose (Chromotek, gtma-20), Pierce Anti-DYKDDDDK Magnetic Agarose (ThermoFisher Scientific, A36797) and IgG beads as an internal control (Binding Control Magnetic Agarose Bead, Chromotek, bmab-20). Immunoprecipitations and inputs were 20-fold diluted and 2.5 μl mixed with 500 nM of each primer and LightCycler 480 Sybr Green I master mix (Roche Applied Science) for qPCR analysis. Products were amplified and fluorescence signals acquired using a LightCycler^®^ 480 detection system. The specificity of amplification products was determined by melting curves. The relative quantification was performed following the ΔΔCt method, and input and IgG values were used to normalize and calculate the % of input. Details for primers used for ChIP-qPCR can be found in Supplemental Table S1.

## RESULTS

### GCN5 positively regulates gene expression by promoting H3K14ac deposition in the 5′ untranslated region of its targets

**Figure 1.**
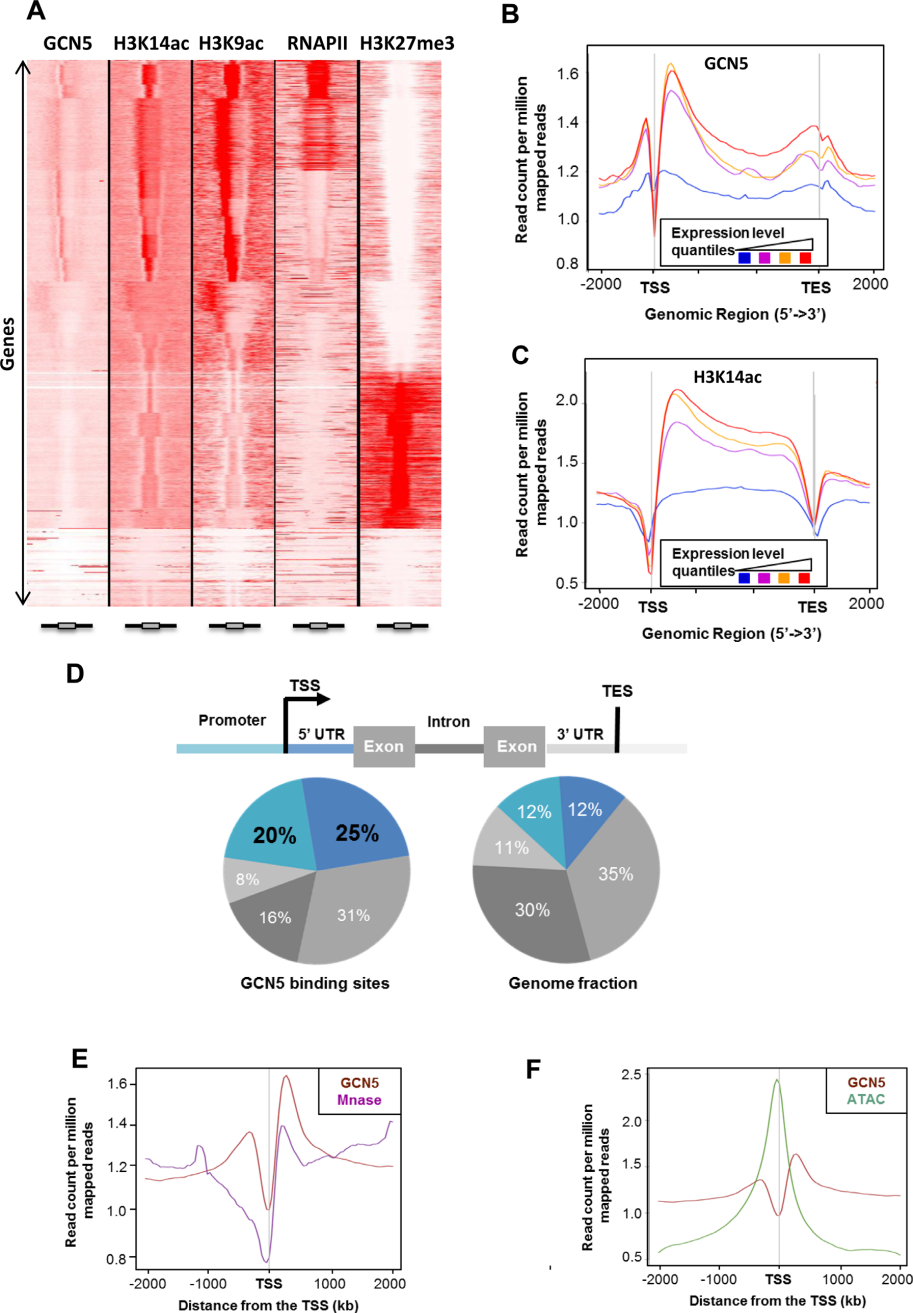
GCN5 binds both promoter and 5′UTR region. (**A**) Association of GCN5 with H3K14ac, H3K9ac, RNAPII and H3K27me3. Comparison of tag density in the region of ±2 kb around ORFs. ChIP-seq were performed on 14-day-old seedlings, *gcn5-1 35S::GCN5-GFP* and WT. Boxes at the bottom of the heatmap represent gene models. (**B**) Average enrichment profile of GCN5 correlates with gene expression levels. Gene expression was categorized in four quantiles from low (blue) to high expression (red). Mean-normalized ChIP-Seq densities of equal bins along the gene and 2 kb region flanking the Transcription Start Site (TSS) and the Transcription End Site (TES) were plotted. Highly expressed genes show higher enrichment for binding of GCN5. (**C**) Average enrichment profile of H3K14ac correlates with gene expression variations. Gene expression was categorized in four quantiles from low (blue) to high expression (red). Mean-normalized ChIP-Seq densities of equal bins along the gene and 2kb region flanking the TSS and the TES were plotted. Highly expressed genes show higher enrichment for H3K14ac. (**D**) Pie chart representation of the distribution of GCN5 peaks identified by ChIP-seq in different genomic regions. The definition of each region is described above. (**E**) Mean profile of GCN5 ChIP-seq and MNase-seq reads density centered on the TSS. Normalization of coverage using spline algorithm was performed over TSS and 2kb flanking regions. (**F**) Merged profiles of GCN5 ChIP-seq and ATAC-seq reads density centered on the TSS. Normalization of coverage using spline algorithm was performed over the TSS and 2kb flanking regions.

GCN5 was previously described as an H3K14 acetyltransferase and is thus considered as a positive regulator of gene expression through interaction with target promoters (14,23,24,33). To study its mode of action at the genome-wide level, we analyzed the distribution of GCN5 and H3K14ac in WT *Arabidopsis* plants through a ChIP assay followed by sequencing (ChIP-seq). To this end, we complemented the *gcn5-1* mutant with a GFP-tagged version of GCN5. We identified a wide repertoire of GCN5 binding peaks corresponding to 8001 genes (Supplemental Table S1, Supplemental Figure S1). As expected from the H3K14ac HAT role of GCN5, its binding showed a strong correlation with H3K14ac peaks (Figure 1A). We combined these ChIP-seq results with transcriptomic data of WT plants and found that both GCN5 binding and H3K14ac levels positively correlate with gene expression (Figure 1B-C and Supplemental Figure S2). Using additional ChIP-seq experiments, we also compared the distribution of GCN5 with H3K9ac and RNAPII binding (the latter dataset previously published (48)), as well as with the repressive mark H3K27me3. As expected from its known role as a positive regulator of gene expression, we observed that GCN5 binding correlates with H3K9ac and RNAPII binding, while it largely anticorrelates with H3K27me3 in WT seedlings (Figure 1A).

We next refined our analysis of the GCN5 mode of action by precisely mapping its binding to different types of genomic regions. After peak annotation, we compared GCN5 binding to the genomic regions of the entire *Arabidopsis* genome and observed an enrichment of GCN5 binding sites on regions upstream of transcription start sites (TSSs), usually corresponding to promoter regions and to 5′ untranslated regions (UTRs) (Figure 1D). The comparison of the GCN5 binding sites with nucleosome distribution and chromatin accessibility, analyzed through MNase-seq and ATAC-seq respectively (datasets that have been previously published (49)), revealed that GCN5 binding correlates partially with nucleosome occupancy. GCN5 binding displays two peaks, one before and one after the TSS (Figure 1B). The peak immediately downstream the TSS matched perfectly histone distribution and H3K14ac enrichment (Figure 1B, E-F), suggesting that this protein is recruited on chromatin through its bromodomain, which recognizes acetylated histones (Figure 1E-F, Supplemental Figures S3 and S4). By contrast, the upstream peak - which does not coincide with H3K14ac enrichment - corresponds to the nucleosome-free region (Figure 1C), suggesting that GCN5 can also be recruited on chromatin independently of acetylated histone recognition. This result is consistent with our previous study showing that GCN5 binding to chromatin can occur in the absence of its bromodomain (36). We therefore performed *de novo* motif discovery on the 5′ UTR and on promoter regions of GCN5 targets using the HOMER software, identifying highly significant motifs on both. Among them, the G-Box consensus sequence (CACGTG) is one of the most represented *cis* elements within the GCN5 peaks (Supplemental Figure S5), consistent with the central role of GCN5 in light responses (23). These data suggest that GCN5 can be recruited to chromatin both through its interaction with specific transcription factors and *via* the direct interaction of its bromodomain with acetylated histones.

**Figure 2.**
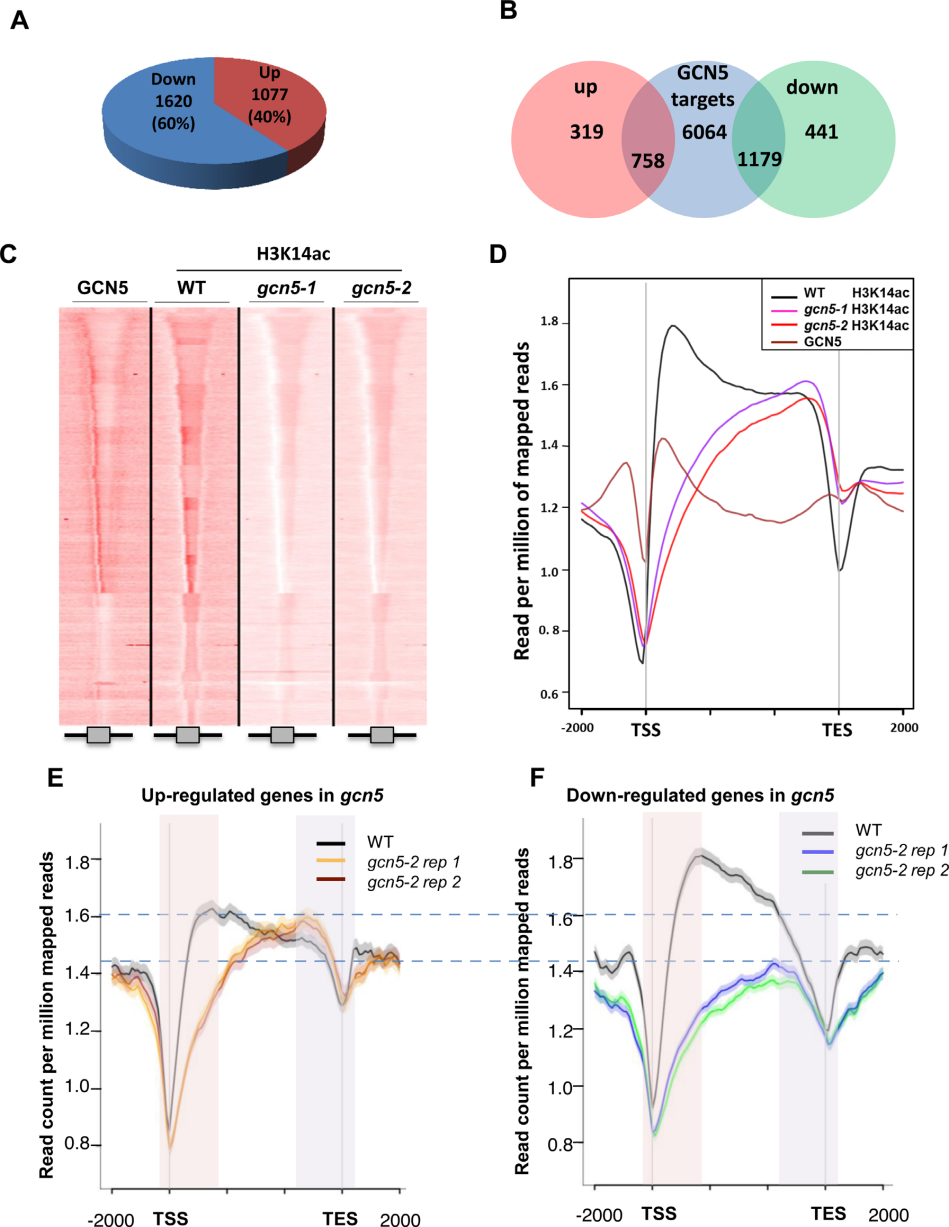
GCN5 can either activate or repress gene expression by controlling H3K14ac distribution on its target genes (**A**) Summary of changes in gene expression observed in *gcn5* mutant. 60% of *gcn5* deregulated genes are down-regulated (1620 genes) whereas 40% are up-regulated (1077 genes). (**B**) Venn diagram representing the overlap between GCN5 targets, identified by ChIP-seq, and the misregulated genes in *gcn5* mutant. (**C**) H3K14ac chromatin profiles in WT, *gcn5-1* and *gcn5-2* mutants compared to GCN5. Comparison of tag density in the region of ±2 kb around ORFs. ChIP-seq were performed on 14-day-old seedlings grown under long day condition conditions. (**D**) Merged profiles of GCN5 and H3K14ac in WT, *gcn5-1* and *gcn5-2* mutants. Mean-normalized ChIP-Seq densities of equal bins along the gene and 2kb region flanking the TSS and the TES were plotted. (**E**) Merged H3K14ac profiles in WT and *gcn5-2* mutant (two biological replicates), restricted to genes that are both GNC5 targets and up-regulated in *gcn5-2* mutant. Mean-normalized ChIP-Seq densities of equal bins along the gene and 2kb region flanking the TSS or the TES were plotted. Shadings highlight 5′ (red) and 3′ (purple) gene end regions. (**F**) Merged H3K14ac profiles in WT and *gcn5-2* mutant (two biological replicates), restricted to genes that are both GNC5 targets and down-regulated in *gcn5-2* mutant. Mean-normalized ChIP-Seq densities of equal bins along the gene and 2kb region flanking the TSS or the TES were plotted. Shadings highlight 5′ (red) and 3′ (purple) gene end regions.

To further confirm the role of GCN5 as an activator of gene expression, we integrated our ChIP-seq data of GCN5 binding sites with RNA-seq data from the *gcn5* mutant (Supplemental Table S1). We observed that the expression of 2697 genes was altered in the *gcn5* mutant (*q*-value < 0.05) when compared to the WT. Among these, 60% were down-regulated and 40% were up-regulated (Figure 2A), consistent with the proposed role of GCN5 as a transcriptional activator (14). In agreement with the described function of GCN5, the majority of down-regulated genes in *gcn5* mutants were identified as direct GCN5 targets (1179 GCN5 target genes, comprising 73% of the down-regulated genes in *gcn5* mutants, Figure 2B). However, to our surprise, 70% of the up-regulated genes in *gcn5* mutants were also found to be GCN5 targets (Figure 2B), suggesting a complex role for this HAT in gene expression, as GCN5 may also act as a transcriptional repressor of specific targets.

### GCN5 can either activate or repress gene expression by controlling H3K14ac distribution on its target genes

To further elucidate whether GCN5 acts both as an activator and repressor of gene expression on different loci, we investigated how it influences genome-wide H3K14ac levels, since it specifically catalyzes the deposition of this mark (24). We analyzed the H3K14ac landscape in the WT and in *gcn5-1* and *gcn5-2* mutants (Figure 2C), identifying 31,651 peaks in the WT and 26 627 peaks in the mutants. We then compared the three ChIP-seq datasets using seqMINER and diffReps, which allow qualitative and quantitative comparisons between a reference set of genomic positions and multiple ChIP-seq datasets. Through this approach, we observed a strong reduction of H3K14ac levels at the 5′ end of genes in the *gcn5-1* and *gcn5-2* mutants compared to the WT (Figure 2D). Surprisingly, we also observed an increase of this mark at the 3′ end of genes in both *gcn5* mutants (Figure 2D), suggesting that GCN5 inversely controls the deposition of this mark at the 5′ and 3′ end of genes.

To further dissect the role of GCN5 in the control of the expression of its targets, we specifically analyzed the H3K14ac profile of the GCN5 target genes and performed this analysis separately for up- and down-regulated targets in *gcn5* mutants (Figure 2E-F and Supplemental Figure S6). This approach discriminated between two contrasting situations: for GCN5 target genes that are down-regulated in *gcn5* mutants, we observed a loss of H3K14ac on the 5′ end of the gene and no significant change in H3K14ac levels on the 3′ end (Figure 2F, Supplemental Figures S6B and S7). By contrast, up-regulated targets were found to present an increase of this mark on their 3′ ends in addition to reduced H3K14ac levels on the 5′ end (Figure 2E, Supplemental Figures S6B and S7). To confirm this observation made on two biological replicates of *gcn5-2* mutant, we performed additional biological replicates of H3K14ac ChIP-seq also in another independent allele, *gcn5-1* (Supplemental Figure S7). Furthermore, genes positively regulated by GCN5 showed a much higher peak of GCN5 binding and H3K14ac levels on their 5′ UTR than genes repressed by GCN5 (Figure 2E-F and Supplemental Figures S6 and S7). Altogether, these results indicate that GCN5 differentially regulates H3K14ac levels and the expression of different sets of genes.

### H3K14ac influences H3K9ac deposition

**Figure 3.**
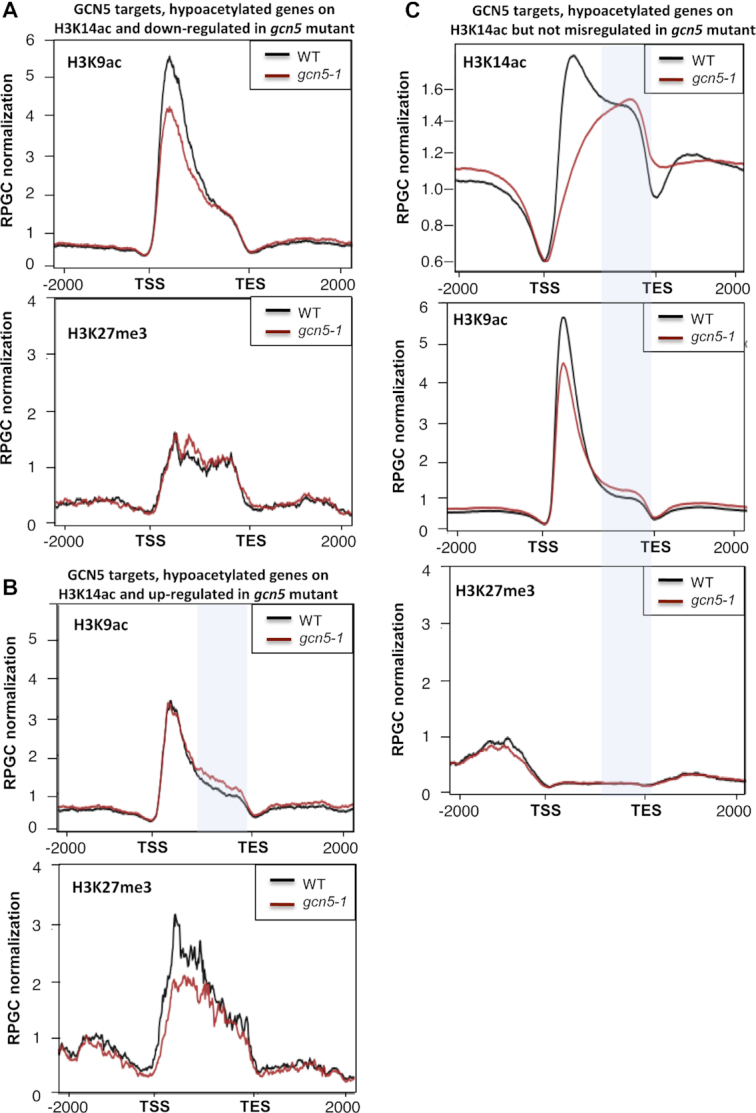
GCN5 influences the deposition of H3K9ac through its action on H3K14ac. (**A**) (top) H3K9ac merged profiles of WT and *gcn5-1* mutant, restricted to genes that are both GNC5 targets and down-regulated in the mutant. (bottom) H3K27me3 merged profiles of WT and *gcn5-1* mutant, restricted to genes that are both GNC5 targets and down-regulated in the mutant. Mean-normalized ChIP-Seq densities of equal bins along the gene and 2kb region flanking the TSS and the TES were plotted. (**B**) (top) H3K9ac merged profiles of WT and *gcn5-1* mutant, restricted to genes that are both GNC5 targets and up-regulated in the mutant. (bottom) H3K27me3 merged profiles of WT and *gcn5-1* mutant, restricted to genes that are both GNC5 targets and up-regulated in the mutant. The blue shading highlights the 3′ end part where H3K14ac hyperacetylation is observed on the same set of genes. Mean-normalized ChIP-Seq densities of equal bins along the gene and 2 kb region flanking the TSS and the TES were plotted. (**C**) (top) H3K14ac merged profiles of WT and *gcn5-1* mutant, restricted to genes that are both GNC5 targets and not differentially expressed in the mutant. (middle) H3K9ac merged profiles of WT and *gcn5-1* mutant, restricted to genes that are both GNC5 targets and not differentially expressed in the mutant. (bottom) H3K27me3 merged profiles of WT and *gcn5-1* mutant, restricted to genes that are both GNC5 targets and not differentially expressed in the mutant. The blue shading highlights the 3′ end part where H3K14ac hyperacetylation is observed. Mean-normalized ChIP-Seq densities of equal bins along the gene and 2kb region flanking the TSS and the TES were plotted.

We next asked whether H3K14ac could influence the levels of other chromatin marks. For this purpose, we chose to test H3K9ac, another activating mark, since we previously showed that the *gcn5* mutation alters the deposition of this mark on the promoters of some specific targets (23). We also evaluated the repressive mark H3K27me3 to test whether GCN5 may antagonize the deposition of this mark by the Polycomb repressive complexes (PRCs), since the GAGA motifs involved in the recruitment of PRCs (50) were found among the over-represented sequences in our motif-enrichment analysis (Supplemental Figure S5). We found that H3K9ac levels correlated with H3K14ac: accumulation of H3K9ac was clearly reduced in the 5′ end of GCN5 target genes that were down-regulated in *gcn5* mutants (Figure 3A and Supplemental Figure S8A) and increased in the 3′ end of targets that were up-regulated in *gcn5* mutants (Figure 3B and Supplemental Figure S8B), suggesting that a molecular crosstalk exists between the deposition of the two marks. Importantly, these changes in H3K9ac were not a consequence of changes in gene expression since they were also observed on GCN5 targets that were not differentially expressed in *gcn5* mutants (Figure 3C and Supplemental Figure S8C). By contrast, we did not observe any increase in H3K27me3 in *gcn5* mutants (Figure 3A-C and Supplemental Figure S8) suggesting that H3K14ac is not a key factor that would prevent H3K27me3 deposition. It is worth noting however, that up-regulated GCN5 targets displayed a slight decrease in H3K27me3 levels (Figure 3C and Supplemental Figure S8B) that might result from their transcriptional activation. Overall, these results suggest that although GCN5-bound regions are enriched in GAGA motifs, GCN5 does not directly influence H3K27me3 deposition but that the deposition of H3K14ac by GCN5 could favor the acetylation of lysine 9 of histone H3.

### GCN5 regulates plant immune responses

**Figure 4.**
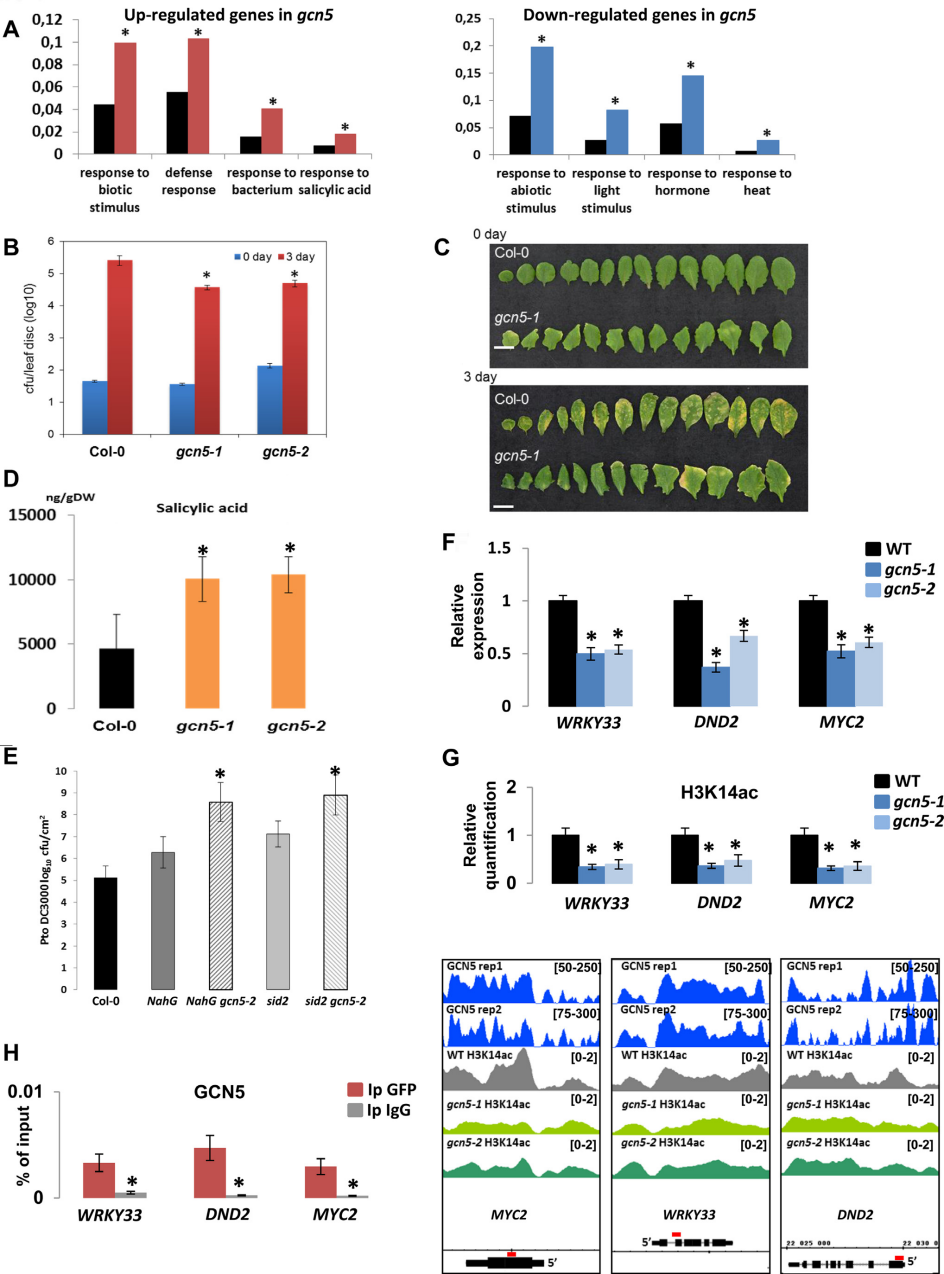
GCN5 regulates plant immune responses through the control of SA homeostasis. (**A**) (left) Gene Ontology analysis of the GCN5 target genes that are up-regulated in *gcn5* mutant compared to WT. The red bars represent the input and the black ones the reference. (right) Gene Ontology analysis of the GCN5 target genes that are down-regulated in *gcn5* mutant compared to WT. The blue bars represent the input and the black ones the reference. Asterisks indicate a statistically significant enrichment of the corresponding gene categories compared to the reference (AgriGO; *t*-test, *P* < 0.05). (**B**) Susceptibility of *gcn5-1* and *gcn5-2* mutants to *Pseudomonas syringae* pv. *tomato* DC3000 was compared to WT Col-0. Bacteria were quantified 2 hrs (day 0) and three days (day 3) after spraying. *gcn5-1 and gcn5-2* mutant plants showed enhanced resistance compared to Col-0. Average values and standard deviations were calculated from three independent experiments. Asterisks indicate statistical significance compared to the WT (*t*-test, *P* < 0.05). (**C**) Image illustrating symptoms development in WT and *gcn5-1* plants after infection with *Pseudomonas syringae* pv. *tomato* DC3000. (**D**) Salicylic acid (SA) levels were determined in WT, *gcn5-1* and *gcn5-2* adult mutant plants in mock condition. Bars represent standard deviation between three biological replicates and asterisks indicate statistical significance compared to the WT (*t*-test, *P* < 0.05). (**E**). Susceptibility of *NahG, NahG gcn5-2, sid2* and *sid2 gcn5-2* mutants to *Pseudomonas syringae* pv. *tomato* DC3000 was compared to WT Col-0. Bacteria were quantified 2 hrs (day 0) and three days (day 3) after spraying. *NahG gcn5-2 and sid2 gcn5-2* mutant plants showed enhanced susceptibility compared to Col-0. Average values and standard deviations were calculated from two independent experiments. Asterisks represent statistical significance compared to the WT (*t*-test, *P* < 0.05). (**F**) Relative expression of SA-related genes in 14-day-old seedlings, WT and *gcn5* mutants. *WRKY33*, *DND2* and *MYC2* expression was extracted from the RNA-seq data. Values are average from three independent replicates. Asterisks represent significant difference with WT (*q* < 0.05). (**G**) (top) Quantification data of the H3K14ac immunoprecipitation results. Chromatin-protein complexes from WT and *gcn5* mutants were immunoprecipitated with H3K14ac specific antibodies. H3K14ac enrichment on the tested regions (*WRKY33*, *DND2* and *MYC2*) was monitored by qPCR. Asterisks indicate significantly different values with respect to the WT (Student's *t*-test, *P* < 0.05). (bottom) Visualization of ChIP-seq results showing GCN5 binding and H3K14ac levels of a few selected genes, *MYC2*, *WRKY33*, *DND2*, that are GCN5 targets and down-regulated in both *gcn5* mutants. The regions highlighted in red above the gene structures were amplified in the ChIP-qPCRs. (**H**). Quantification data of GCN5 immunoprecipitation results. Chromatin-protein complexes from *gcn5-1 35S::GCN5-GFP* mutant were immunoprecipitated (Ip) with antibodies specific for GFP or IgG. GCN5 enrichment on the tested regions (*WRKY33*, *DND2* and *MYC2*) was monitored by qPCR. Asterisks indicate significantly different values with respect to the WT (Student's *t*-test, *P* < 0.05).

To apprehend the biological relevance of GCN5 epigenetic regulations, we performed an analysis of Gene Ontology (GO) terms amongst genes differentially expressed in *gcn5* mutants. Down-regulated genes showed a significant enrichment in GO categories related to responses to light and heat, consistent with the previously described role of GCN5 as an activator of these pathways (23,33,36) (Figure 4A). By contrast, up-regulated genes were enriched in categories related to defense and salicylic acid (SA) responses (Figure 4A) and included genes such as *PCC1* (*Pathogen and Circadian Controlled 1*), *AtHIR1* (*Hypersensitive-Induced Response protein 1*) and *FRK1* (*Flg22-induced Receptor-like Kinase 1*) (Supplemental Figure S9), suggesting that GCN5 acts as a repressor of SA-mediated immunity.

Since SA-related genes are differentially expressed in *gcn5* mutants, we tested the direct relevance of GCN5 during biotic stress by testing the resistance of *gcn5* mutants to the bacterial pathogen *Pseudomonas syringae* pv. *tomato* DC3000. For this experiment, we sprayed a bacterial suspension on 1-month-old plants and quantified the bacterial population 0- and 3-days post-inoculation. When comparing the bacterial colony-forming units (CFU) per leaf area of *gcn5-1* and *gcn5-2* mutants and the WT, a statistically significant decrease in the bacterial count was observed in both mutants (Figure 4B), as well as a reduction in the severity of symptoms (Figure 4C). To confirm that the observed phenotype is due to the *gcn5* mutation and that the construct used is functional, we tested the resistance of our *gcn5* mutant complemented line (*gcn5-1 35S::GCN5-GFP*) to the bacterial pathogen *Pst* DC3000 and observed no difference between WT and the complemented line (Supplemental Figure S10). These results indicate that GCN5 acts as a negative regulator of immune responses to hemibiotrophic pathogens.

To determine whether the enhanced resistance of *gcn5* mutants was the result of changes in hormonal regulation, as indicated by the differential regulation of SA-related genes, we quantified SA accumulation in these lines and found that both mutant lines display a significant increase in the SA levels under control conditions compared to WT plants (Figure 4D), suggesting that the observed increase in pathogen resistance in *gcn5* mutants occurs through an up-regulation of the SA pathway. To confirm this hypothesis, the *gcn5-1* mutant was crossed with an *NahG*-overexpressing transgenic line (expressing a bacterial enzyme that degrades SA (38)), and the *sid2* mutant (deficient in *ICS1*, a key enzyme for SA biosynthesis (37)). As expected, the overexpression of *NahG* or the mutation of *ICS1* in the *gcn5-2* mutant background abolished its enhanced resistance and led to increased sensitivity compared to the WT, as was observed in the *NahG* overexpression line and *sid2* mutant (Figure 4E), confirming that the increased resistance of the *gcn5* mutants to *Pst* DC3000 is a consequence of increased SA accumulation. We noticed an enhanced sensitivity of the double mutants compared to the single *NahG* and *sid2* mutants, probably due to the additive pleiotropic effects of each of these single mutations.

Because GCN5 acts mainly as a positive regulator of gene expression, we searched for genes involved in the control of SA accumulation amongst the down-regulated target genes in *gcn5* mutants. We found that *MYC2*, *DEFENSE NO DEATH2 (DND2)* and *WRKY33*, which are negatives regulators of SA biosynthesis (51–53), are down-regulated in the two independent *gcn5* mutant alleles (Figure 4F), revealing a new putative role of GCN5 in plant responses to biotic stress. Consistently, ChIP-qPCR experiments using either complemented *gcn5* mutant lines, *gcn5-1* 35S::*GCN5-GFP* or *gcn5-2 GCN5p*::*GCN5-FLAG*, confirmed that these genes are direct GCN5 targets and that H3K14ac levels are reduced at these loci in the *gcn5* mutants (Figure 4G-H and Supplemental Figure S11). These results show that GCN5 plays a new role in plant immunity through the regulation of SA homeostasis.

## DISCUSSION

In this study, we explored the molecular function of GCN5 at genome-wide scale to define its role in the regulation of transcription and histone mark deposition. GCN5 was found to colocalize with the H3K14ac mark genome wide (Figure 1A) and its binding is associated with high expression levels (Figure 1B), as is expected from its role as a HAT. However, the deposition of H3K14ac was found to be limited to the gene bodies, with a significant enrichment in their 5′ ends (Figure 1C), while GCN5 binding was also observed upstream of the TSS, on the promoters of its targets. This latter result indicates that GCN5 can bind to acetylated histones through its bromodomain as well as to nucleosome-free regions, supporting previous observations that the mutation of the bromodomain does not affect the binding of GCN5 to most of its target promoters (36). This suggests that GCN5 recruitment to most of its targets occurs though its interaction with other proteins and complexes, such as ADA2, which interacts with GCN5 through its HAT domain and stimulates the acetylation of nucleosomal histones, a process that GCN5 cannot carry out alone (54). The SAGA complex in plants is composed of more than 20 proteins, some of which constitute the SPT module, implicated in SAGA recruitment to chromatin (22). In yeast and mammals, the SAGA complex is recruited to its target loci through the interaction of TRA1 (or its mammalian ortholog TRRAP) with specific transcriptional activators, and it is thought that this protein may serve as a scaffold for the recruitment of SAGA to chromatin. Even though there are two TRA1 homologues encoded in the *Arabidopsis* genome (TRA1A and TRA1B), these proteins remain to be characterized in depth, as well as the detailed molecular mechanisms by which SAGA is recruited to specific loci.

Furthermore, the SAGA TAF module, which is highly conserved within the plant kingdom, has been reported to contribute to the activation of specific gene sets by SAGA, since several *TAF* genes have been shown to display tissue- and developmental stage-specific expression patterns (55). Notably, we found that GCN5 binds preferentially to certain DNA motifs, some of which have been previously related to responses to environmental stimuli, such as GAGA repeats, that have been reported to be targets of PRC1 and PRC2, protein complexes essential for genome-wide transcriptional silencing through the addition of repressive H3K27me3 and ubiquitin marks (50). However, GCN5 binding is largely anticorrelated with H3K27me3 occupancy (Figure 1A); hence, GCN5 may possibly compete with PRCs for the binding of these latter motifs to control the equilibrium between transcriptionally active and silenced loci.

### GCN5 prevents H3K14 acetylation at the 3′ end of its targets and regulates H3K9ac levels

Previous studies showed that GCN5 deposits the H3K14ac mark (24) and positively regulates other histone acetylation marks, such as H3K9ac and H3K27ac (23). However, in the current study we found that GCN5 loss-of-function leads to increased H3K14ac levels in the 3′ end of some of its targets (Figure 2D), indicating that GCN5 also negatively regulates the deposition of this mark in these regions. Since GCN5 is a H3K14 HAT itself, it can be predicted that it antagonizes the activity of other HATs that perform H3K14 acetylation through a mechanism that remains to be elucidated. Interestingly, ELO3/ELP3, a protein from the GCN5 family and a subunit of the Elongator complex, has been previously shown to positively regulate H3K14ac in the 3′ end of its targets to promote their expression in response to auxin signals (56). Thus, it could be proposed that GCN5 dampens ELP3 activity through a yet unknown mechanism; however, this hypothesis should be addressed experimentally by assessing H3K14ac levels in the double *elo3 gcn5* mutant. We found a clear relationship between the gain of 3′ H3K14ac in the *gcn5* mutant and the transcriptional up-regulation of several genes in this mutant (Figure 2E), which suggests that this 3′ hyperacetylation contributes to the previously reported ambivalent role of GCN5 in transcription regulation (29,36). Interestingly, we observed that three genes, *CNGC12*, *AtHIR1* and *WRKY57*, which are GCN5 targets and up-regulated in both *gcn5* mutants, display an increase of H3K14ac mark on their 3′ ends (Supplemental Figure S12). Given that these genes are involved in pathogen resistance (57–59), it suggests that GCN5 could control negatively the deposition of H3K14ac at the 3′ end of some stress-regulated genes.

**Figure 5.**
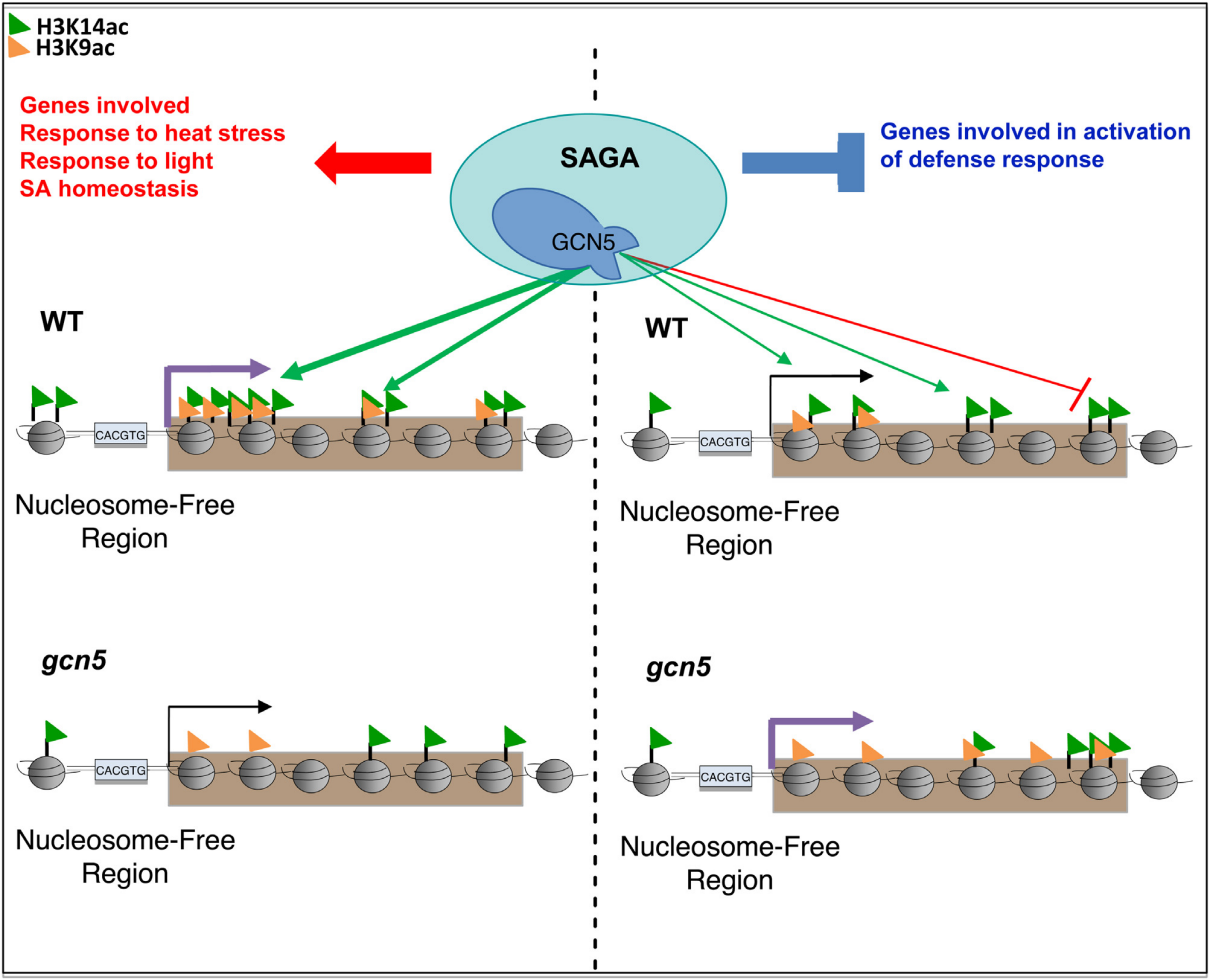
GCN5 is a master regulator of responses to environmental stimuli. GCN5 promotes the deposition of H3K14ac on the 5′ end of its target genes involved in heat stress response, response to light or inhibition of SA accumulation and functions as a positive regulator of these genes. By contrast it antagonizes H3K14 acetylation of genes involved in biotic stress response and thereby represses their expression. In this way, GCN5 may function to keep a balance between biotic and abiotic stress responses.

It was previously proposed that GCN5 acetylates lysines 9 and 14 of histone 3, since the *gcn5* mutation induces a reduction in both marks on loci involved in specific physiological processes (31–33,35). However, by using radioactively labeled acetyl groups, it was shown that the AtGCN5 HAT activity is specific for H3K14ac, and the acetylation of H3K9 has been attributed to HATs with broad specificity, such as HAC1, HAC5 and HAC12 (24). In this study, we provide genome-wide evidence indicating that GCN5 positively regulates the deposition of H3K9ac (Figure 5); nevertheless, since GCN5 does not perform this acetylation itself, it could be hypothesized that this protein (and/or H3K14 acetylation) facilitates the recruitment of other HATs that conduct H3K9 acetylation, or prevent the activity of HDACs such as HDA19, which perform the removal of this histone mark (60).

### GCN5 is a master regulator of responses to environmental stimuli

Histone modifications are major regulatory mechanisms controlling gene expression. It is thus not surprising that an increasing number of publications report the role of these chromatin modifications in plant immunity, including histone acetylation (61–63). In this study, we show that GCN5 is a negative regulator of SA accumulation and SA-mediated immunity in *Arabidopsis* (Figure 4), adding this protein to the growing list of epigenomic regulators involved in plant defense responses. Various HDACs have been associated with the transcriptional reprogramming that occurs during immune responses; for instance, in a previous publication, we found that flagellin perception results in dramatic genome-wide H3K9ac changes, and that the HDAC HD2B is a major contributor of this process (64). Furthermore, HDA19 has also been reported to be involved in defense, and similar to GCN5, it has been described as a repressor of the SA-mediated branch of immunity (65). On the other hand, some HATs have also been described as positive regulators of immunity, including AtHAC1 (66) and various components of the Elongator complex in different plant species (67–71). In the case of GCN5, the *gcn5* mutant presents a significant up-regulation of immune-related genes, which coincides with the repression and hypoacetylation of various loci encoding for repressors of SA-mediated immunity, including *DND2*, *WRKY33* and *MYC2* (Figure 4F-H). We therefore hypothesize that GCN5 negatively regulates SA-mediated immunity, at least partially, by positively regulating the expression of immune repressors, such as the previously mentioned loci; however, this remains to be experimentally assessed.

Beyond the role of GCN5 in the regulation of immunity-related genes, we found that down-regulated genes in the *gcn5* mutant were enriched in loci involved in responses to abiotic stimuli, such as light and heat, consistent with the previous report of GCN5 as a crucial element in the activation of light- and heat-responsive genes (23,33,36). GCN5 function in response to temperature is not limited to heat stress, since the *gcn5* mutant has been reported to display a lower and slower accumulation of *Cold Regulated* (*COR*) gene transcripts when compared to the WT in response to cold temperature (29). These results are in favor of a general role of *Arabidopsis* GCN5 as a major regulator of responses to diverse biotic and abiotic stimuli, functioning as an integrator of diverse physiological processes mediating development and adaptation to the environment.

## DATA AVAILABILITY

ChIP-seq raw data have been deposited to the Gene Expression Omnibus (GEO) database under accession number GSE137474.

## Supplementary Material

gkaa369_Supplemental_Files
