# Supplementary material for: GCN5 modulates salicylic acid homeostasis by regulating H3K14ac levels at the 5′ and 3′ ends of its target genes
Source: Nucleic Acids Res. 2020 May 12;48(11):5953–66. doi: 10.1093/nar/gkaa369 (PMC7293002; doi:10.1093/nar/gkaa369)
Supplement: gkaa369_Supplemental_Files [file gkaa369_supplemental_files.zip › GCN5_Supplemental_Figures_27042020.pdf]

Supplemental Figure S1

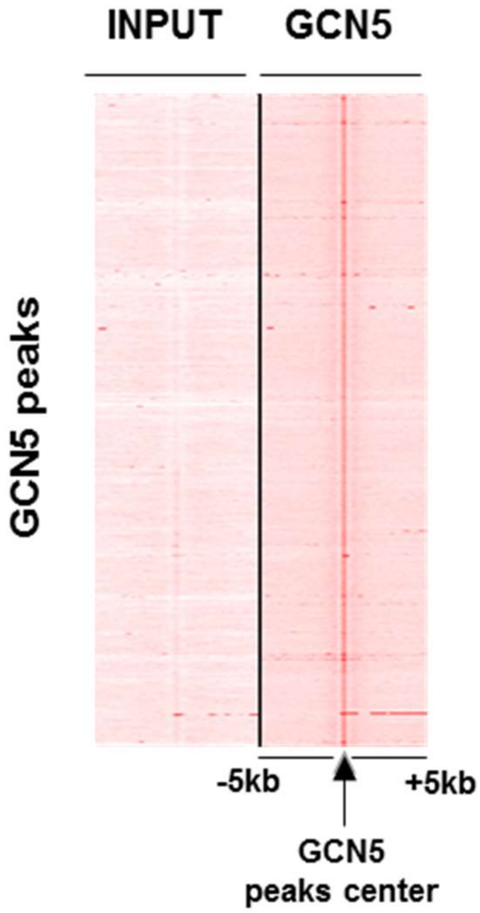

Supplemental Figure S1: Comparison between Input and GCN5 immunoprecipitation tag density in the region of ±5 kb around the GCN5 peaks. ChIP-seq was performed on 14-day-old *gcn5-1 35S::GCN5-GFP* seedlings using GFP antibody.

Supplemental Figure S2

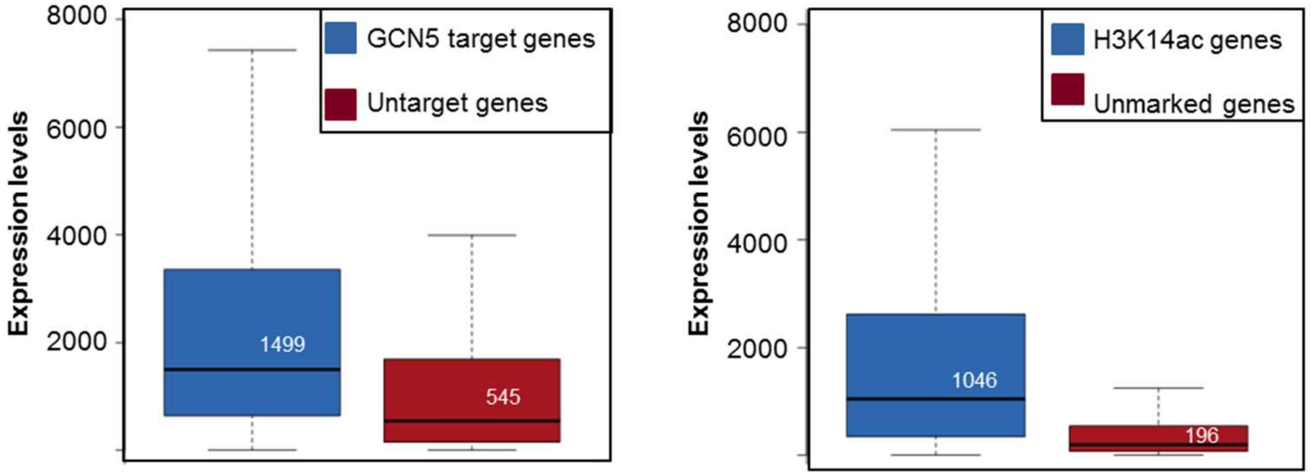

Supplemental Figure S2: (left) Boxplot showing comparison of expression levels of GCN5 target genes and non-target genes in Col-0. GCN5 target genes show higher expression levels. (right) Boxplot showing comparison of expression levels of H3K14ac marked genes and non-marked genes in Col-0. H3K14ac marked genes show higher expression levels.

Supplemental Figure S3

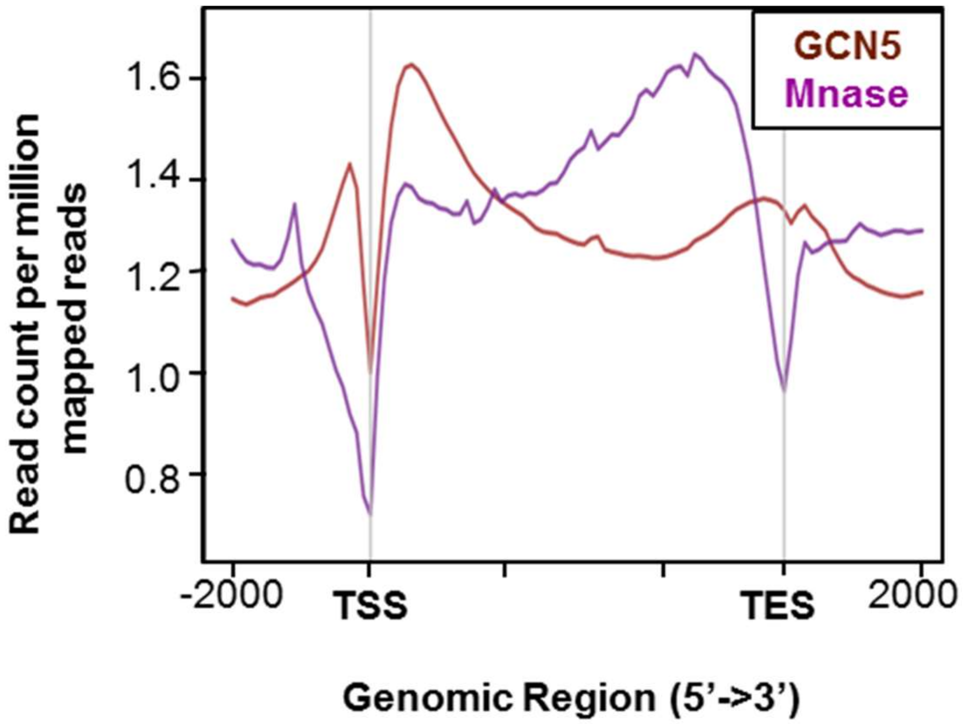

Supplemental Figure S3: Mean profile of GCN5 ChIP-seq and WT MNase-seq reads density with respect to a gene model from Transcription Start Site (TSS) to Transcription End Site (TES). Normalization of coverage using spline algorithm was performed over the genes and flanking 2kb region.

Supplemental Figure S4

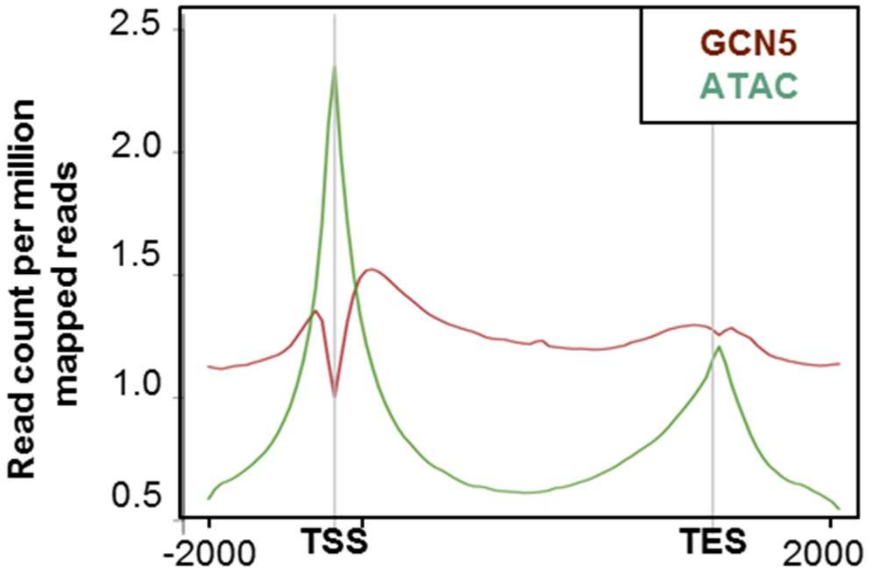

Supplemental Figure S4: Mean profile of GCN5 ChIP-seq and WT ATAC-seq reads density with respect to a gene model from Transcription Start Site (TSS) to Transcription End Site (TES). Normalization of coverage using spline algorithm was performed over the genes and flanking 2kb region.

Supplemental Figure S5

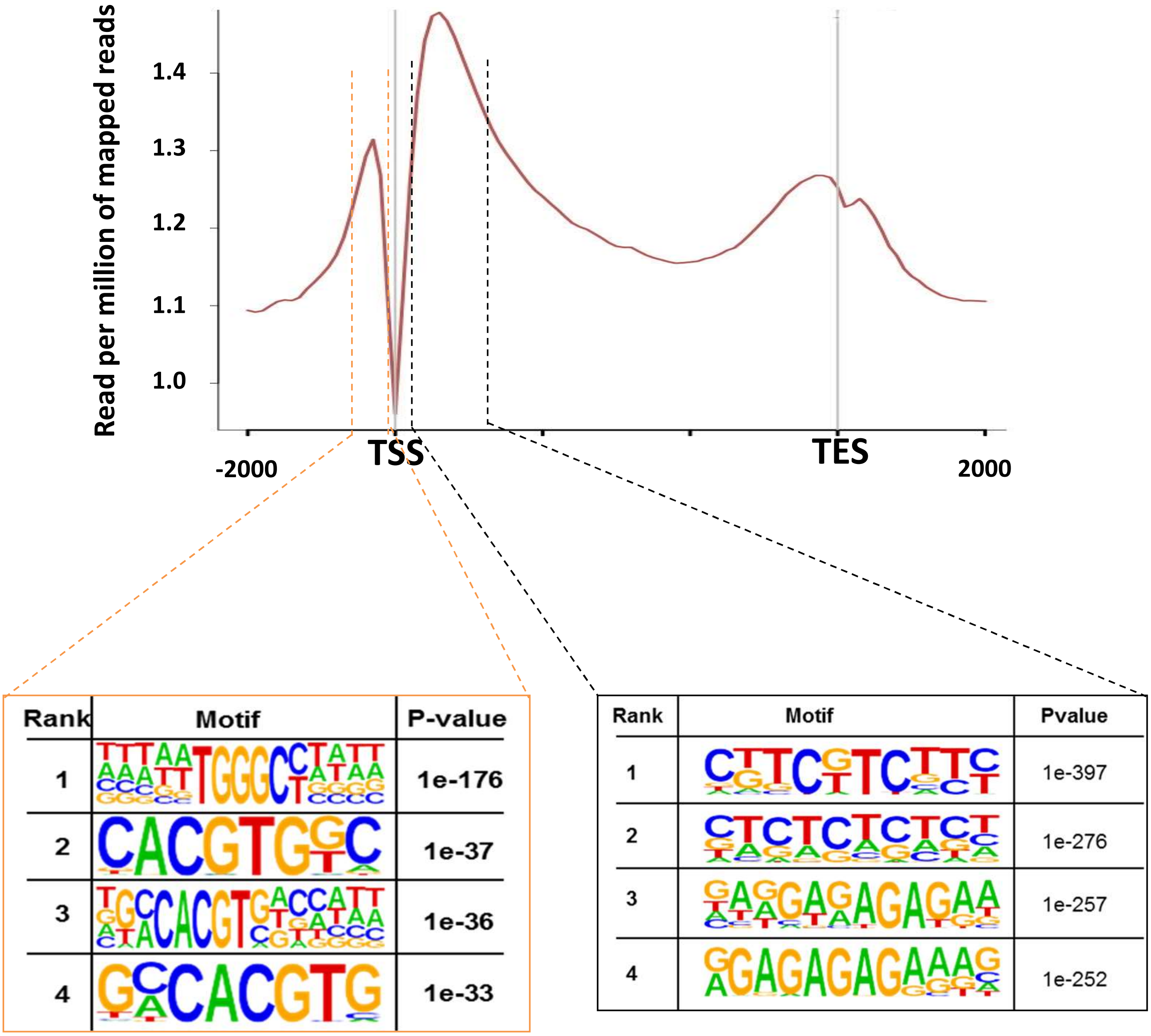

Supplemental Figure S5: List of motifs found based on all GCN5 target genes. Sequence motifs were detected from HOMER analysis of GCN5 target genes on both promoter (left) and 5'UTR (right) region.

Supplemental Figure S6

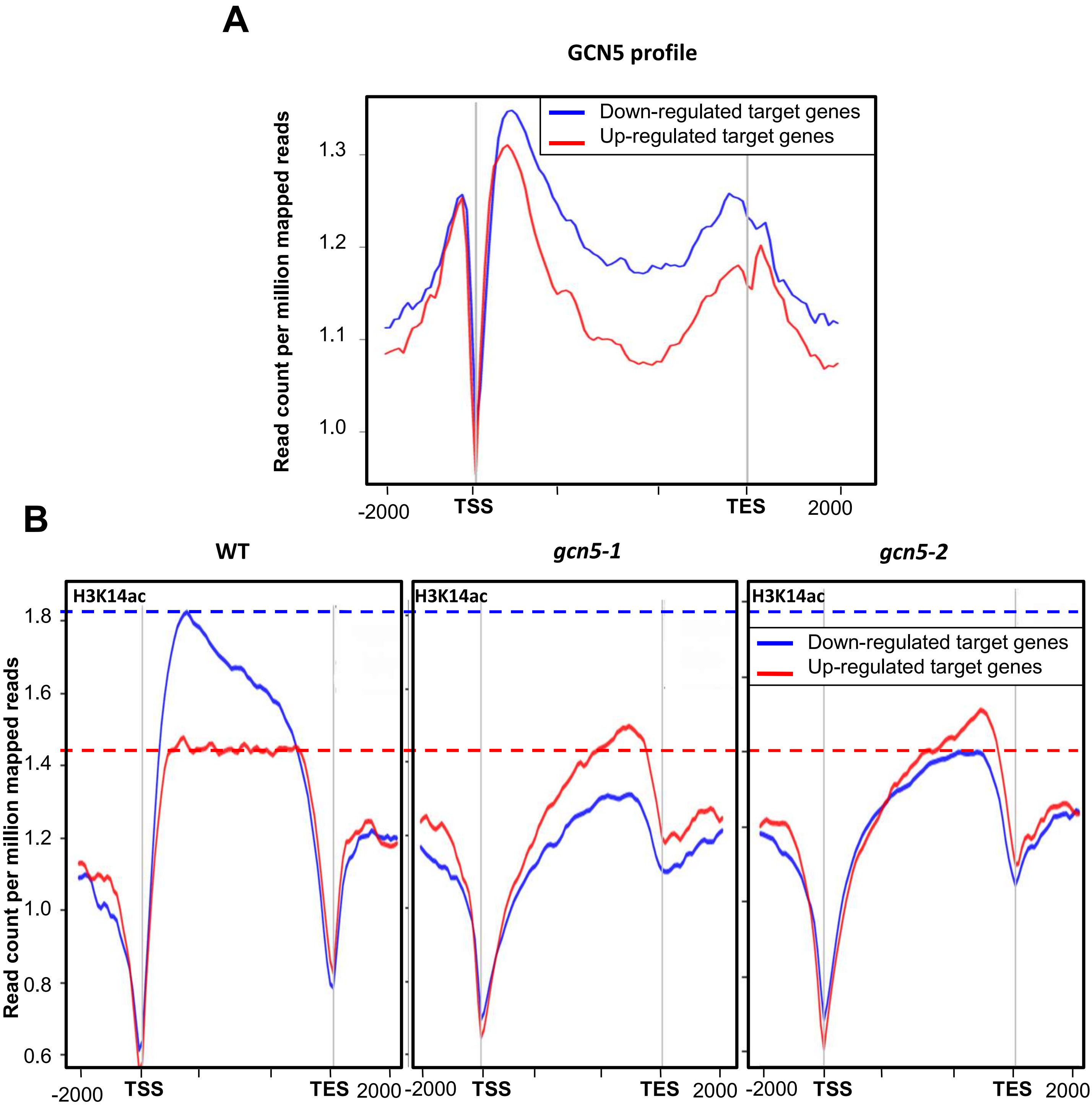

Supplemental Figure S6:

(A) Average tag density profile of GCN5 on targeted and differentially regulated genes in *gcn5* mutants. Mean-normalized ChIP-seq densities of equal bins along the gene and 2kb region flanking the TSS or the TES were plotted. Up- and down-regulated genes are categorized with a q-value cutoff of 0.05.

(B) Average tag density profile of H3K14ac on GCN5 targeted and differentially regulated genes in WT, *gcn5-1* and *gcn5-2* mutants. Mean-normalized ChIP-seq densities of equal bins along the gene and 2kb region flanking the TSS or the TES were plotted. Up- and down-regulated genes are categorized with a q-value cutoff of 0.05.

Supplemental Figure S7

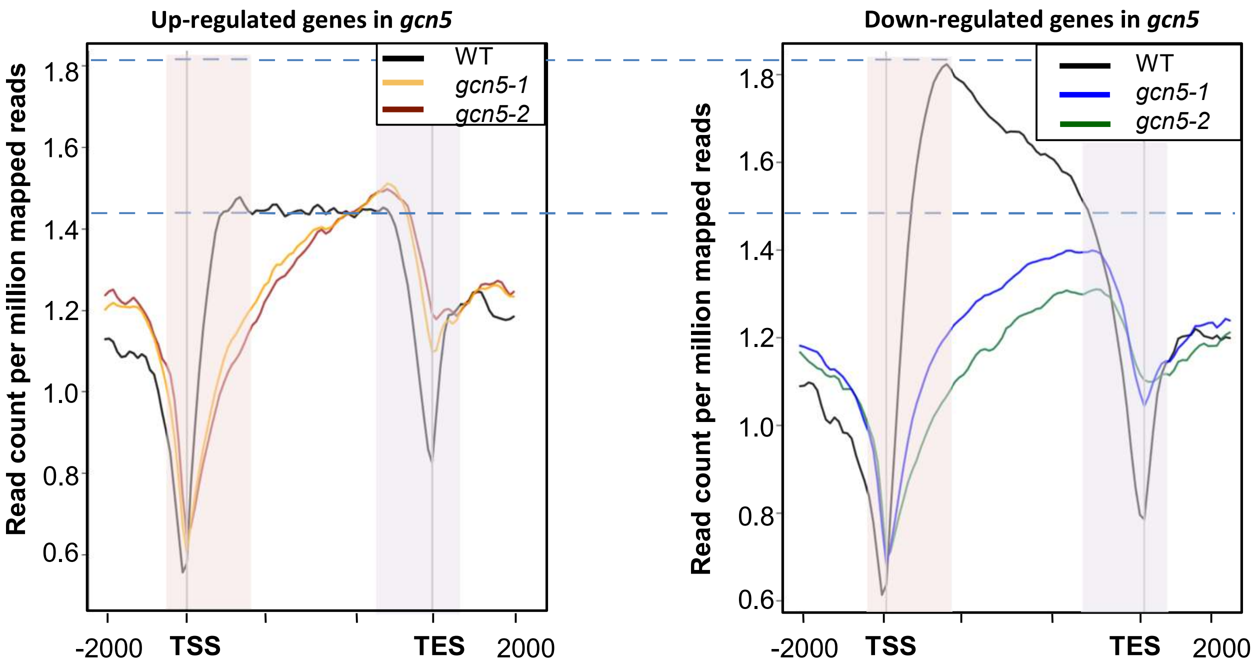

Supplemental Figure S7:
Merged H3K14ac profiles in WT, *gcn5-1* and *gcn5-2* mutants, restricted to genes that are both GNC5 targets and up-regulated in *gcn5* mutants (left) or genes that are both GNC5 targets and down-regulated in *gcn5* mutants (right). Mean-normalized ChIP-Seq densities of equal bins along the gene and 2kb region flanking the TSS or the TES were plotted. Shadings highlight the 5' (red) and 3' (purple) gene end regions.

Supplemental Figure S8

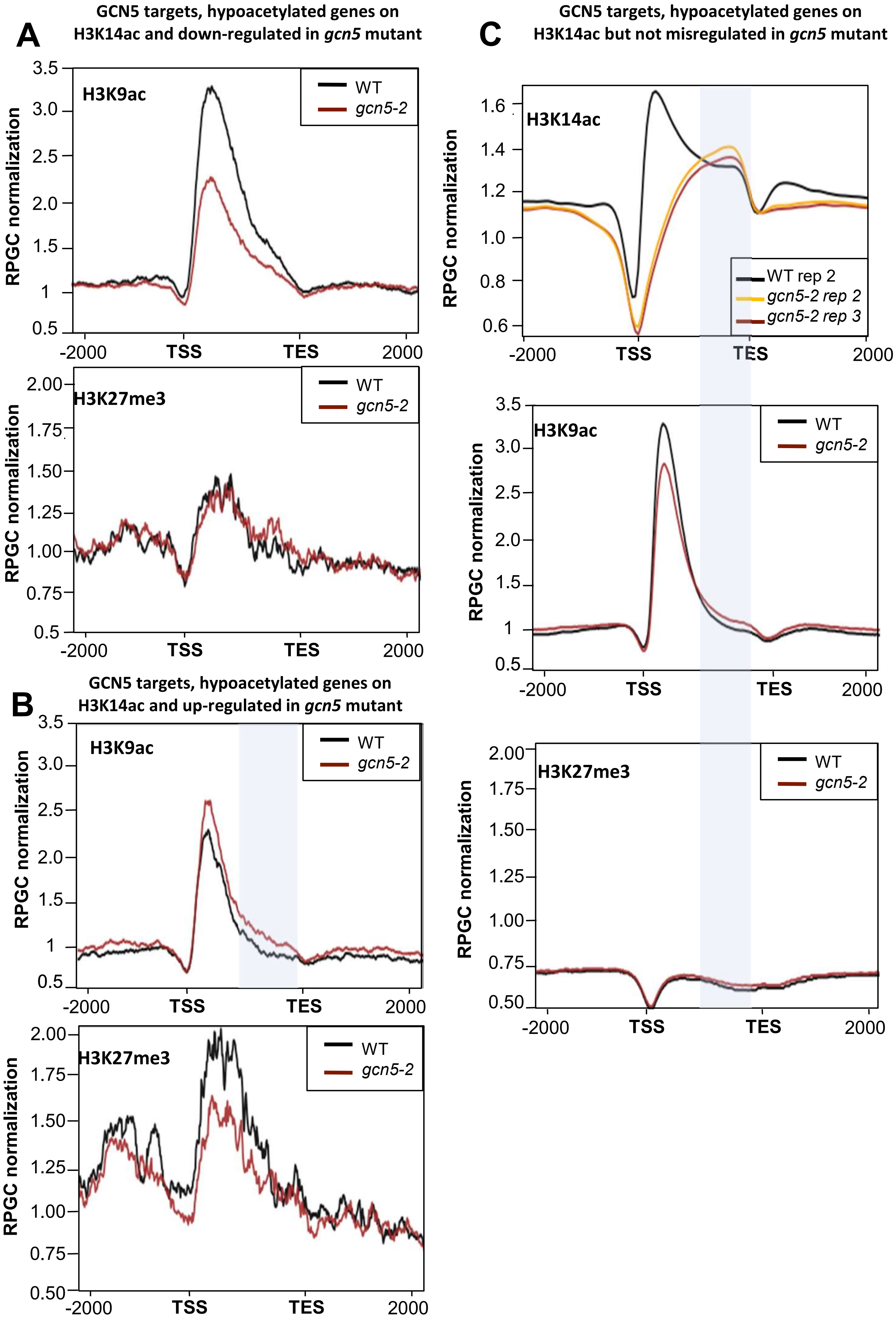

Supplemental Figure S8:

(A) (top) H3K9ac merged profiles of WT and *gcn5-2* mutant, restricted to genes that are both GNC5 targets and down-regulated in the mutant. (bottom) H3K27me3 merged profiles of WT and *gcn5-2* mutant, restricted to genes that are both GNC5 targets and down-regulated in the mutant. Mean-normalized ChIP-Seq densities of equal bins along the gene and 2kb region flanking the TSS and the TES were plotted.

(B) (top) H3K9ac merged profiles of WT and *gcn5-2* mutant, restricted to genes that are both GNC5 targets and up-regulated in the mutant. (bottom) H3K27me3 merged profiles of WT and *gcn5-2* mutant, restricted to genes that are both GNC5 targets and up-regulated in the mutant. The blue box highlights the 3' end part where a hyperacetylation of H3K14ac is observed on the same set of genes. Mean-normalized ChIP-Seq densities of equal bins along the gene and 2kb region flanking the TSS and the TES were plotted.

(C) (top) H3K14ac merged profiles of WT and *gcn5-2* mutant (including two biological replicates for the mutant), restricted to genes that are GNC5 targets but not misregulated in the mutant. (middle) H3K9ac merged profiles of WT and *gcn5-2* mutant, restricted to genes that are GNC5 targets but not misregulated in the mutant. (bottom) H3K27me3 merged profiles of WT and *gcn5-2* mutant, restricted to genes that are GNC5 targets but not misregulated in the mutant. The blue box highlights the 3' end part where a hyperacetylation of H3K14ac is observed. Mean-normalized ChIP-Seq densities of equal bins along the gene and 2kb region flanking the TSS and the TES were plotted.

Supplemental Figure S9

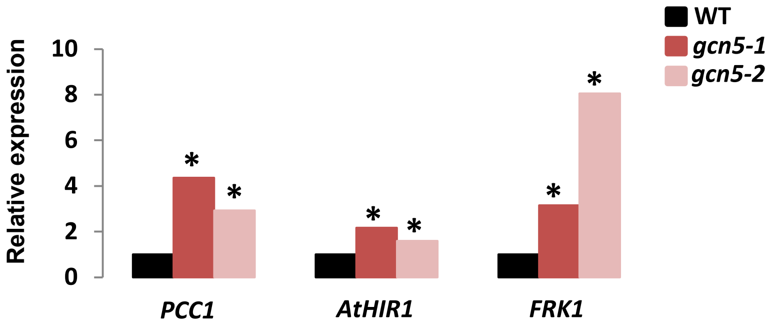

Supplemental Figure S9: Relative expression of *PCC1*, *AtHIR1* and *FRK1* genes, in 14-day-old seedlings, WT and *gcn5* mutants. Data were extracted from the RNA-seq dataset. Values are average from three independent replicates. Asterisks represent significant difference with the WT ($q < 0.05$).

Supplemental Figure S10

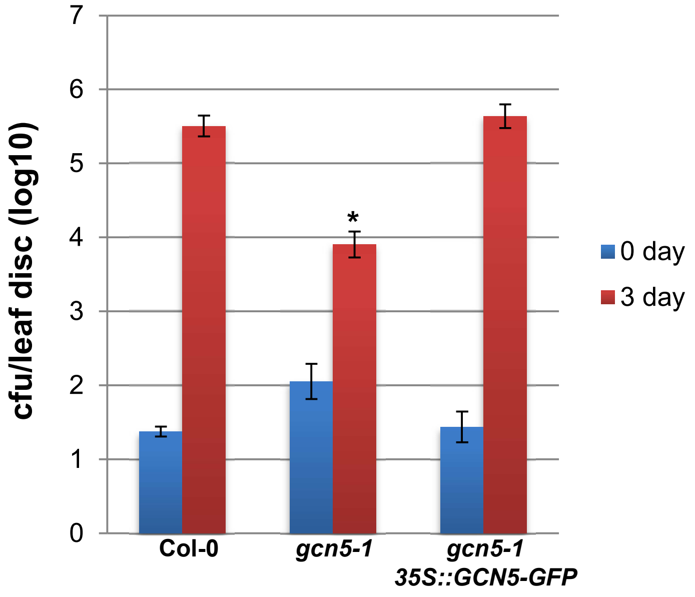

Supplemental Figure S10: Susceptibility of *gcn5* complemented mutant (*gcn5-1 35S::GCN5-GFP*) to *Pseudomonas syringae* pv. *tomato* DC3000 compared to WT Col-0 and *gcn5-1* mutant. Bacteria were quantified 2 hrs (day 0) and three days (day 3) after spraying. *gcn5-1 35S::GCN5-GFP* mutant plants showed similar susceptibility to Col-0. Average values and standard deviations were calculated from two independent experiments. Asterisks represents statistical significance compared to the WT (t-test, $p<0.05$).

Supplemental Figure S11

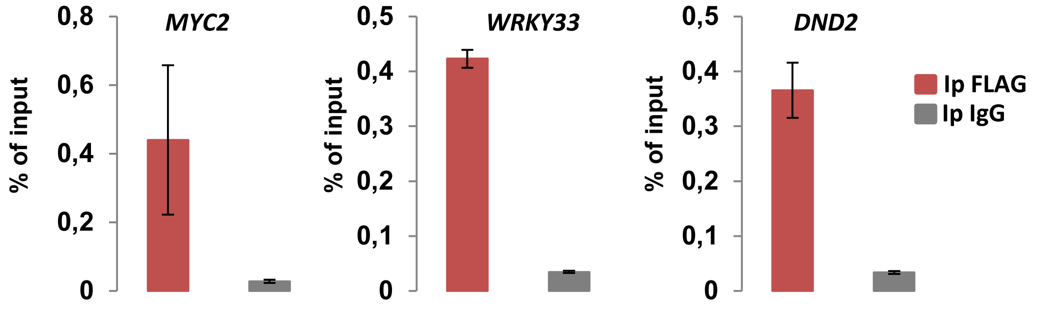

Supplemental Figure S11: Quantification data of GCN5 immunoprecipitation results. Chromatin-protein complexes from *gcn5-2 GCN5p::GCN5-FLAG* mutant were immunoprecipitated (Ip) with antibodies specific for FLAG or IgG. GCN5 enrichment on the tested regions (*MYC2*, *WRKY33* and *DND2*) was monitored by qPCR.

Supplemental Figure S12

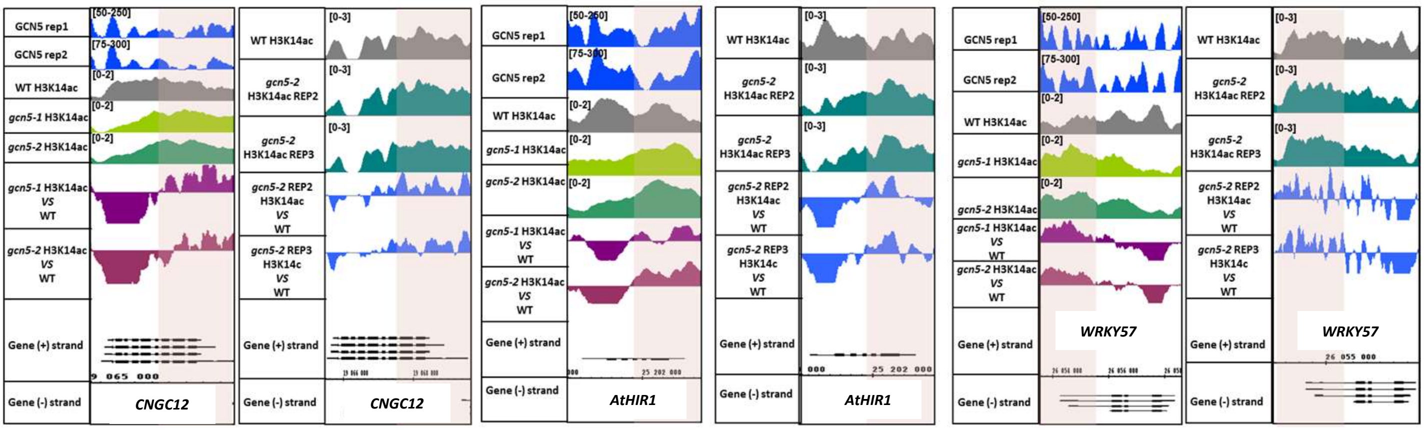

Supplemental Figure S12: Visualization of ChIP-seq results showing GCN5 binding and H3K14ac levels of selected defense-related genes, *CNGC12*, *AtHIR1*, *WRKY57*, that are GCN5 targets and up-regulated in both *gcn5* mutants. For each gene, left and right panels present different biological replicates.
